# Evaluation of a co‐delivered training package for community mental health professionals on service user‐ and carer‐involved care planning

**DOI:** 10.1111/jpm.12378

**Published:** 2017-04-27

**Authors:** A. C. Grundy, L. Walker, O. Meade, C. Fraser, L. Cree, P. Bee, K. Lovell, P. Callaghan

**Affiliations:** ^1^ School of Health Sciences University of Nottingham Nottingham UK; ^2^ Health Sciences Research, School of Nursing, Midwifery and Social Work University of Manchester Manchester UK; ^3^ Mental Health Nursing School of Health Sciences, University of Nottingham Nottingham UK

**Keywords:** nursing education, practice development, user involvement

## Abstract

**What is known on the subject?:**

- There is consistent evidence that service users and carers feel marginalized in the process of mental health care planning.
- Mental health professionals have identified ongoing training needs in relation to involving service users and carers in care planning.
- There is limited research on the acceptability of training packages for mental health professionals which involve service users and carers as co‐facilitators.

**What does this paper add to existing knowledge?:**

- A co‐produced and co‐delivered training package on service user‐ and carer‐involved care planning was acceptable to mental health professionals.
- Aspects of the training that were particularly valued were the co‐production model, small group discussion and the opportunity for reflective practice.
- The organizational context of care planning may need more consideration in future training models.

**What are the implications for practice?:**

- Mental health nurses using co‐production models of delivering training to other mental health professionals can be confident that such initiatives will be warmly welcomed, acceptable and engaging.
- On the basis of the results reported here, we encourage mental health nurses to use co‐production approaches more often.
- Further research will show how clinically effective this training is in improving outcomes for service users and carers.

**Abstract:**

**Background**

There is limited evidence for the acceptability of training for mental health professionals on service user‐ and carer‐involved care planning.

**Aim**

To investigate the acceptability of a co‐delivered, two‐day training intervention on service user‐ and carer‐involved care planning.

**Methods**

Community mental health professionals were invited to complete the Training Acceptability Rating Scale post‐training. Responses to the quantitative items were summarized using descriptive statistics (Miles, 2013), and qualitative responses were coded using content analysis (Weber, 1990).

**Results**

Of 350 trainees, 310 completed the questionnaire. The trainees rated the training favourably (median overall TARS scores = 56/63; median ‘acceptability’ score = 34/36; median ‘perceived impact’ score = 22/27). There were six qualitative themes: the value of the co‐production model; time to reflect on practice; delivery preferences; comprehensiveness of content; need to consider organizational context; and emotional response.

**Discussion**

The training was found to be acceptable and comprehensive with participants valuing the co‐production model. Individual differences were apparent in terms of delivery preferences and emotional reactions. There may be a need to further address the organizational context of care planning in future training.

**Implications for practice**

Mental health nurses should use co‐production models of continuing professional development training that involve service users and carers as co‐facilitators.

## Background

Mental health care planning is a process whereby any issues raised in an assessment (such as problems, strengths, goals and planned interventions) are put into a plan of care, which is then implemented and regularly reviewed (Hall & Callaghan 2008). International mental health policy initiatives dictate that this process becomes a collaborative one involving the service user, any close family member or carer, and associated mental health professionals (Healthcare Commission 2008a, Commonwealth of Australia 2009, Department of Health 2011, World Health Organisation 2012). Whilst staff are primarily concerned with the *outcome* of care planning (i.e., a signed care plan), service users are more concerned with the *process* of care planning, and particularly the user–clinician relationship (Bee *et al*. 2015a, Grundy *et al*. 2015, Simpson *et al*. 2016). Although substantial evidence suggests that service users are sufficiently motivated to collaborate in the care planning process, poor information exchange and insufficient opportunities for shared decision‐making pose major barriers to this (Bee *et al*. 2015a). Historically, service user involvement has been typically limited to the retrospective endorsement of professional care decisions (McDermott 1998), leaving service users feeling marginalized and disempowered (Grundy *et al*. 2015) and carers feeling disregarded (Cree *et al*. 2015). Lack of service user involvement occurs in both inpatient and community settings (Healthcare Commission 2008a,b, CQC 2009) and across different care trajectories and professional roles (Bee *et al*. 2008, Goss *et al*. 2008).

Mental health professionals have themselves identified ongoing training requirements for staff on service user‐ and carer‐involved care planning. In a qualitative study of professional perspectives on service user‐ and carer‐involved care planning, staff recognized that training in person‐centred communication and relational skills in the context of care planning would be helpful (Bee *et al*. 2015b). They acknowledged the lack of standardized care planning training in pre‐ and post‐registration courses and felt that current continuing professional development (CPD) training was ‘ad hoc' and infrequently evaluated (Bee *et al*. 2015b). The majority of staff consulted felt they lacked an awareness of effective models for engaging service users in care planning discussions and wanted to revisit foundational listening and engagement skills (Bee *et al*. 2015b). Staff also wanted training in user‐involved care planning to address the organizational context in which care planning occurs (Bee *et al*. 2015b).

Importantly, staff also wanted training in understanding engagement and involvement in care planning from the service user perspective and indicated they would welcome a specialized training programme that involved service users and carers (Bee *et al*. 2015b). Recent reviews (Repper & Breeze 2007, Morgan & Jones 2009, Terry 2012, Happell *et al*. 2014) have highlighted the policy imperative of involving service users and carers in healthcare education, and there is tentative evidence that this involvement in training enhances professionals' skills in the manner prioritized by service users (Repper & Breeze 2007). There is also limited evidence to suggest that students and service users both feel that they benefit from service user involvement in educational programmes (Morgan & Jones 2009), but because such training programmes have rarely been formally evaluated (Terry 2012, Happell *et al*. 2014), it is unclear what mental health professionals value about CPD training and about co‐production models of training in particular. Moreover, whilst the most common form of user involvement in mental healthcare education is via the service user sharing personal narratives to ‘tell their stories’ (Repper & Breeze 2007), other models of user involvement (such as co‐facilitation) have rarely been evaluated (Happell *et al*. 2014). Therefore, whilst clinicians recognize the need for a new training package in user‐involved care planning and feel that they would welcome some form of co‐production model, such a package and approach needs to be rigorously evaluated to see whether it is appropriate and acceptable and thus whether staff feel that they are likely to try to implement new learning.

The current study is part of a wider programme of work funded by the National Institute for Health Research (NIHR) exploring service user‐ and carer‐involved care planning (EQUIP: Enhancing the quality of user‐involved care planning in mental health services) (Bower *et al*. 2015). This research is currently trialling the clinical and cost‐effectiveness of a co‐delivered (by clinicians/academics, service users and carers) training programme for community‐based mental health professionals to enhance service user and carer involvement in care planning. The current study as part of the EQUIP trial explores whether this co‐produced, co‐delivered, specialized training programme was acceptable to those mental health professionals who attended the training.

## Methods

### Aim of the study

The aim of the study was to investigate the acceptability to community mental health professionals of a co‐delivered training intervention on involving service users and carers in mental health care planning.

### EQUIP training course

Following the synthesis of data from previous work (Bee *et al*. 2015a,b, Brooks *et al*. 2015 Cree *et al*. 2015, Grundy *et al*. 2015), the research team produced a training manual and presentation slides for a two‐day training course on enhancing service user and carer involvement in mental health care planning for community‐based mental health professionals.

Each of the two days' training began at 09:30 and finished by 16:30. Training was held at team bases, or other NHS Trust training venues, or on university premises, depending on team preference. Lunch and refreshments were provided throughout the day. Trainees were given a pack with handouts of the presentation slides. Trainees were asked to bring one anonymized care plan per team.

On day one, following introductions, the team explained that the training course was part of the EQUIP cluster randomized controlled trial (Bower *et al*. 2015). The first topic was focussed on understanding care planning in terms of the policy rhetoric and the reality of care planning on the ground. This was followed by an interactive presentation on what is now known about service user‐ and carer‐involved care planning, based on a recent realist review (Bee *et al*. 2015a). This led onto a session on what good care planning looks like from the service user, carer, and professional perspectives. After lunch, trainees explored engagement and communication skills and finished the first day looking at explaining care planning terms and processes.

Day two began with user‐centred assessment, exploring issues around ‘risk’ and ‘safety’, before moving onto co‐producing summary and formulation statements. The afternoon was spent looking at developing aspirational goals and exploring what shared decision‐making looks like and concluded by thinking about user‐involved implementation and reviewing of care planning.

Following the training, trainees were emailed a package of resources to supplement learning and teams were also offered up to six hours of clinical supervision. All the EQUIP training materials are freely available by either contacting the lead author or via the EQUIP web site: http://research.bmh.manchester.ac.uk/equip.

### Trainers

At each session, the training team consisted of an academic researcher with a clinical background in mental health nursing (PC or KL) and one or two service users (AG, DB or LW) and, where possible, a carer (LC). The service users and the carer were recruited from either the study team (from the original grant co‐applicants) or from the programme's service user and carer advisory group (SUCAG). Following a brief interview to check suitability, nine trainees attended a four‐day ‘train the trainers’ course (the content and acceptability of which are reported elsewhere, Fraser *et al*. 2017), which gave some teaching theory and teaching practice, and concluded by going through the actual training manual and slides. Six of those trained went on to co‐deliver the training course.

### Delivery

The two‐day EQUIP training intervention consisted of interactive presentations, audio–visual clips, small group exercises, skills practice exercises (including role play) and live demonstrations of good practice, and included working with anonymized care plans or anonymized examples from professionals' caseloads. The team wanted to move away from the ‘sharing personal stories’ model of user/carer ‘involvement’ in delivering training; thus, whilst the academic researcher was the lead facilitator, the service users and the carer facilitated group work, shared both positive and negative experiences of care planning, and shared ideas around good and poor practice with the wider group throughout the two days. The EQUIP training was thus designed to be a co‐produced and co‐delivered training resource.

### Recruitment of community mental health teams into the EQUIP trial

In the EQUIP trial, the study team recruited 36 teams across 10 NHS Trusts to participate in a trial to test the clinical and cost‐effectiveness of the training in enhancing service user and carer involvement in care planning. Meetings were held with team managers and, where possible, staff to facilitate engagement and understanding of the trial. Teams were made aware that they would be either allocated to receive the EQUIP training in care planning or allocated to the control condition, where they would continue with their usual care. Teams were informed that 80% of staff designated as ‘care coordinators’ (i.e. those with a caseload) would need to commit to attend the two days' training if randomized to the intervention. We offered teams a choice of training dates and the option of training as a whole team or training in two halves to minimize service disruption.

### Participants

Participants came from five Trusts in the North of England and five Trusts from the Midlands. Eighteen teams received the training intervention, 9 teams from the North and 9 teams from the Midlands. Attendance at each two‐day session ranged from 4 to 39 trainees (mean 19.44), and in total, 350 completed the training. Overall, the teams consisted of 304 care coordinators, across a wide spectrum of professional roles, the majority of whom were community mental health nurses (*n* = 186). We did not train any psychiatrists, although they were invited to attend. In addition, we trained 46 team members who did not have a care planning caseload. Trainee role profiles are summarized in Table 1.

**Table 1:** Trainee role profiles (*n* = 350)

Care coordinator status	Breakdown by job role	*n*
Care coordinators (*n* = 304)	Community Mental Health Nurses	186
	Occupational Therapists	47
	Social Workers	47
	Team or Assistant Team Managers	9
	Psychologists	6
	Support Workers	4
	Resettlement Workers	2
	Approved Mental Health Professionals	2
	Assistant Practitioners	2
	Clinical Leads (role unknown)	4
Non care coordinators (*n* = 46)	Students	13
	Support Workers	7
	Community Mental Health Nurses	7
	Nursing Assistants	5
	Community Care Officers	3
	Social Workers	3
	Occupational Therapists	2
	Psychological Well‐being Practitioner	1
	Team Mangers (profession unknown)	5

There were 249 women and 101 men. A total of 307 trainees attended both days; 27 people attended day one, but not day two; and 16 people did not attend day one, but did attend day two. Thus, 323 people could have completed the anonymized evaluation at the end of the second day's training. A total of 310 participants actually completed the evaluation.

### Evaluation tool

The Training Acceptability Rating Scale (TARS‐1: Davis *et al*. 1989, TARS‐2: Milne & Noone 1996 pp. 140–141) was used to evaluate the attendees' acceptability of the EQUIP two‐day training course. The first section (TARS‐1) consists of six self‐report items which assess training ‘appropriateness’ or ‘acceptability’ (covering general acceptability, perceived effectiveness, negative side effects, appropriateness, consistency and social validity). Each of the six items is rated on a six‐point Likert scale, ranging from ‘strongly disagree’ (score 1) to ‘strongly agree’ (score 6). TARS‐1 has good test–re‐test reliability (*r *=* *0.83 *P *< 0.01) and internal consistency (0.99) (Davis *et al*. 1989). The second section (TARS‐2) assesses the attendees’ overall impressions of the impact of the teaching process and its outcomes and consists of nine items, rated on a four‐point scale from ‘not at all’ (score 0) to ‘a great deal’ (score 3). Whilst the reliability of TARS‐2 has never been psychometrically assessed, it has repeatedly demonstrated good face and concurrent validity (Carpenter *et al*. 2007).

Questions 1–6 were summed to calculate an overall acceptability score (possible range 6–36), and questions 7–15 were summed to calculate an overall perceived impact score (possible range 0–27). An overall TARS score was calculated by summing the responses to questions 1–15 (possible range 6–63) (Myles & Milne 2004, Milne *et al*., 2007).

TARS‐2 concludes with three open‐ended questions asking about the ‘most helpful’ part of the training, any ‘recommended changes’ and ‘any other comments’.

### Data collection

TARS evaluation data were collected at the end of the final session on day two of the EQUIP training. All completed questionnaires were anonymous.

### Ethical considerations

The completion of the TARS constituted an evaluation, akin to a service evaluation, of a training course for mental health professionals and thus did not require ethical approval. England distinguishes between research as defined by the Frascati definition (Organisation for Economic Cooperation and Development [OECD], 2015) and thus requiring ethical approval and service evaluation and audit that do not require ethical approval (NHS Health Research Authority [HRA], 2016). However, the following considerations were in place: there was a protocol in place for the training programme and evaluation; the completion of the TARS after the training programme was completely voluntary, and finally, the completed measures were anonymous.

### Data analysis

Quantitative analysis of the TARS results was conducted by generating descriptive statistics (Miles 2013) in SPSS version 21. The open‐ended comments were analysed using content analysis, a qualitative method that can classify open‐ended text into categories that represent similar meanings (Weber 1990) and identify trends in the data via the quantification of specific words or themes. Qualitative responses to the three open‐ended questions on the TARS‐2 were inputted into the NVIVO version 11 software management programme and analysed for key themes (Fereday & Muir‐Cochrane 2006). The analysis sub‐team consisted of two service users (AG, LW) and an academic researcher (OM). All comments were read and open‐coded for meaning initially by the lead author and subsequently independently checked by the other two members of the sub‐team. Codes with similar or related meaning were aggregated into themes. We quantified content at the level of the emerging theme. The use of NVIVO allowed the researchers to identify codes and themes and also record the frequency of theme re‐occurrence across all participant responses.

### Findings

#### Quantitative results

The TARS results are detailed in Table 2.

**Table 2:** TARS scores descriptive statistics

Question/domain (possible score range)	*n*	Median	Inter‐quartile range	Range
1. General acceptability (1–6)	309	6	5–6	1–6
2. Perceived effectiveness (1–6)	307	6	5–6	1–6
3. Negative side effects (1–6)	295	6	5–6	1–6
4. Inappropriateness (1–6)	303	6	5–6	1–6
5. Consistency (1–6)	310	6	5–6	1–6
6. Social validity (1–6)	307	6	5–6	1–6
7. Did the training improve your understanding? (0–3)	310	2	2–3	0–3
8. Did the training help you to develop skills? (0–3)	307	2	1–3	0–3
9. Has the training made you more confident? (0–3)	310	2	1–2	0–3
10. Do you expect to make use of what you learnt in the training? (0–3)	306	2	2–3	0–3
11. How competent were those who led the training? (0–3)	309	3	3–3	1–3
12. In an overall, general sense, how satisfied are you with the training? (0–3)	308	3	2–3	0–3
13. Did the training cover the topics it set out to cover? (0–3)	310	3	2–3	0–3
14. Did those who led the training sessions relate to the group effectively? (0–3)	310	3	3–3	1–3
15. Were the leaders motivating? (0–3)	309	3	2–3	0–3
Total ‘acceptability’ Q1–6 (1–36)	289	34	31–36	6–36
Total ‘perceived impact’ Q7–15 (0–27)	301	22	19–25	4–27
Total TARS Q1–15 (6–63)	283	56	51–61	24–63

As demonstrated in Table 2, the scores showed high levels of satisfaction with the training overall and with the acceptability and perceived impact of the training.

For each individual question on the acceptability subscale, there was a median score of 6 (out of a possible range of 0–6). The majority of participants ‘strongly agreed’ that the training was generally acceptable (61.2%), effective/beneficial (58.6%), appropriate (64.7%) and consistent with good practice (73.9%). The majority of participants also ‘strongly agreed’ that the training would not harm clients (74.9%), and they approved of the training (58.3%).

The questions on the perceived impact subscale had a possible score range 0–3. For questions 11–15, the median score was 3, and for questions 7–10, the median was lower at 2. The majority of participants answered ‘a great deal’ to questions 11–15 related to: how competent the course leaders were (79.6%); their satisfaction with the training (54.5%); how well the training covered the course topics intended (57.1%); how the leaders related to the training group (81.9%); and how motivating the leaders were (74.4%). However, the most frequent response to questions 7–10 was ‘quite a lot’. These questions asked whether the training: improved understanding (45.5%); helped them to develop skills (44.6%); increased confidence (42.9%); and would be used by them in future (41.8%).

#### Qualitative results

The results from the three open‐ended questions on the TARS‐2 are here presented under six predominant themes: the value of the co‐production model; time to reflect on practice; delivery preferences; comprehensiveness of content; need to consider organizational context; and emotional response. Of the 310 completed TARS, 300 completed the open‐ended questions; 10 of those received had no responses to the open‐ended questions. Quotations are reported here as they were written by respondents.

### The value of the co‐production model

The co‐production model is key to the EQUIP training intervention, and 102 participants commented on the value of the service user and carer contribution to the training, in terms of the value of their shared experiences, perspectives and insights, and appreciating them as facilitators. One participant commented ‘having service users and carers participating and sharing stories first hand made it all the more meaningful’. Another said that service user input gave ‘meat to the bones of the research outcomes’. Only one participant felt that at times the comments were ‘a little one sided (e.g. extreme examples of poor communication between service users and service)’. However, one participant appreciated the opportunity ‘to test things out’ with the carer, and two participants commented that the involvement helped them put themselves ‘in service user's shoes'. One trainee wanted them to participate in group tasks more actively; another wanted even more service user and carer involvement overall.

### Time to reflect on practice

Participants appreciated the opportunity to take ‘time out’ to reflect on practice (*n* = 50). One commented that the training ‘provided headspace to discuss and learn/reflect on effective care planning’. Eight people commented that they would implement what they have learnt in practice. For example, one person said that ‘I will carry through my training and refer back to frequently’. Three people said that the training would actually change their practice. For example, one participant said ‘the training promoted motivation and encouragement to change clinical practice, promoting service user involvement’. Eight people commented that the training was pertinent to their practice, one participant described the course as ‘relevant training that can be put into practice’, and another that it addresses ‘the issues we face’.

### Delivery preferences

Group discussion was frequently commented on as a helpful part of the training. Participants appreciated the interaction in discussion groups generally (*n* = 28), and in particular, they valued sharing practice with colleagues (*n* = 8), listening to colleagues’ ideas and methods of working (*n* = 5), and obtaining feedback from facilitators (*n* = 1). One participant commented that they really valued ‘being in a supportive group’, and another that it was just good ‘spending time with the team’. Three people wanted more group work, two people commented that it would be good to move people around and change groups, and one person suggested mixing up the facilitators as well. The use of role‐play in the small groups received a mixed reception, with 18 people commenting that it was helpful (‘role plays in small groups very effective in showing different approaches, role modelling, reflecting on what you might say’) and 15 people commenting that they found it unhelpful or would scrap it (‘less reliability on role play, this approach is not helpful in increasing confidence’). A minority suggested a shorter course (*n* = 6) would be preferable. However, three felt that more time was needed. Two people suggested that more audio–visual would be helpful, and one said that the resources were ‘excellent’. Finally, 25 people commented on the training environment, in terms of improving the venue or the hospitality; for example, 3 people commented that they wanted training ‘nearer to team base’, whereas two people wanted training away from the ‘distractions’ of the team base.

### Comprehensiveness of content

Eighty‐eight people commented that they would make no changes to the training content, and 15 explicitly stating that they found both days helpful. Eleven people commented that the training gave a good overview of care planning, four that it was a helpful ‘refresher’ course, and ten mentioned that it promoted client‐centred working. In terms of the particular topics that trainees found most helpful, 17 people found writing summary statements of a service user's condition/situation helpful, in particular one person commented that ‘writing problem statements from the service user perspective’ was helpful, and four commented on using the first person (‘I’ statements) in describing an individual's needs or problems. Seventeen participants found it helpful to explore goal setting, with ‘less emphasis on SMART goals’ and more ‘aspirational goals’. One participant said that they would ‘help service user make own goals in their words’. Fourteen participants found it helpful to explore risk assessment. For example, one participant found it helpful ‘understanding the impact upon service users’ of professional use of language (i.e. risk)’, and 4 people commented that it was helpful thinking about ‘safety’ rather than ‘risk’. One participant reported that assessment could be cut from the programme, and another that there could be less focus on it. However, another trainee reported that the training did not adequately address issues of ‘risk management’.

Four participants suggested new topics, and these were material on writing care plans; for example, one person commented ‘don't feel I'm better prepared to write care plans’, and another wanted ‘more on how to actually write care plans’ particularly around the issue of writing care plans in the first person. One participant reported that more issues around confidentiality should have been covered in the training, saying ‘include a little more re: confidentiality issues, carers/service users when to share and when not to share info’. Another suggestion for improvement (*n* = 15) was that concrete examples of good and poor care plans would have enhanced the training. Four people found the case studies helpful, for example ‘I found the case studies particularly helpful in terms of reflection on my practice’. Three people wanted some examples of older people's mental health needs, and three wanted more community‐based examples as they felt that some of the examples were not relevant to their caseloads. One person wanted more opportunity to reflect on their own caseloads and another to reflect on their care plans.

### Need to consider organizational context

In regard to improving the training, 15 participants stated that the specific organizational context in which care planning occurs should have been factored more into the course. One participant reflected that: ‘I think we've identified issues that we were already aware of prior to the training – that most of the reasons service users have a negative experience are directly related to service or system problems which is greater than the individuals who work directly with the service users. Therefore this training should be given to those that make systems decisions and changes to the system/culture/process of practice’. Another participant reported that the training presented more of an ‘ideal world’. In contrast, another trainee found helpful the ‘idea that organizations are dysfunctional’ and felt that that had been addressed. Five people commented on the lack of psychiatrists at the training, summed up by this comment ‘very disappointed by the lack of medics in the group – NONE!!’ Two people reported that managers and senior managers should be in attendance, and another commented that a wider pool of team members should be in attendance.

### Emotional response

A minority concluded that the training was not helpful at all (*n* = 6). Three participants commented that the training felt ‘negative’. One said ‘It's things we do already and seemed pointless. At times felt like professional banging and saying we're doing it wrong’. Another commented that they ‘found some training patronising’. Furthermore, another said ‘I have felt quite deflated during the training’. For two people, this was explicitly tied to the presentation of the evidence base from a previous realist review (Bee *et al*. 2015a), which highlights the relational and organizational barriers to involving service users and carers in care planning. Others, however, commented that the course was ‘inspirational’ (*n* = 2) and ‘motivating’ (*n* = 2), that it gave ‘positive messages’ (*n* = 2), that it was delivered ‘gently’ (*n* = 1), and that they ‘didn't feel judged’ (*n* = 1) and that they had ‘no fear of saying the wrong thing’ (*n* = 1).

## Discussion

Post‐registration, CPD training for mental health professionals has rarely been formally evaluated; thus, it has been unclear what clinicians value about such training packages (Bee *et al*. 2015b). Similarly, co‐production approaches to training, with service users and carers as co‐facilitators, have until now rarely been evaluated (Terry 2012). Mental health professionals have reported that they want training in understanding engagement and involvement in care planning from the service user perspective and that they would welcome a training package based on a co‐production model (Bee *et al*. 2015b). The EQUIP training intervention was devised to meet these demands and to respond to consistent evidence of the marginalization of service users and carers in care planning (Bee *et al*. 2015a). This study explored whether a co‐produced, co‐delivered, specialized training programme in service user‐ and carer‐involved care planning is acceptable to mental health professionals attending the training.

The overall TARS scores demonstrated high levels of satisfaction with the training generally and with the acceptability and impact of the training for mental health professionals. Improved understanding, developing skills, increased confidence, and future use of the training were rated quite highly (‘quite a lot’) but not as positively as all the other perceived impact items (‘a great deal’). This could be that participants already understood care planning, and that they felt their skills were already good (some did describe the course as a ‘refresher’, or that it consolidated their outlook) or that these would take time to consolidate after the training. As a result of these scores, the team decided to explore these issues more fully in the subsequent clinical supervision sessions that were offered to teams.

This study sheds light on what mental health professionals appreciate about the inclusion of service users in co‐facilitating training. In line with previous reviews (Repper & Breeze 2007, Morgan & Jones 2009, Terry 2012, Happell *et al*. 2014), mental health professionals valued service users and the carer sharing their experiences, but the current study shows that staff appreciated them as training facilitators and valued their ideas around good and poor practice in relation to care planning in particular. This approach enabled clinicians to better understand engagement and involvement in care planning from the user/carer perspectives. This study lends credence to the suggestion that involving service users in healthcare training enhances professionals’ skills in the manner prioritized by service users (Repper & Breeze 2007). The EQUIP cluster randomized controlled trial will test whether the training actually enhances clinical practice (Bower *et al*. 2015).

The study also highlights what in particular clinicians’ value about CPD training, which has until now rarely been explored (Repper & Breeze 2007, Terry 2012). The EQUIP two‐day interactive training package focussed on how to engage with, explain to, and involve service users and carers in the whole process of care planning, including assessment and formulation, planning and goal setting, implementation and review. The majority of staff wanted to think about all of these topics and appreciated the range of teaching methods and especially small group interaction with colleagues and with the facilitators. In particular, staff appreciated the opportunity to take ‘time out’ to reflect upon their practice and to spend time with their team. Therefore, team‐based training, with interaction in small groups, is thus an acceptable and engaging format for learning for mental health professionals.

In terms of improving the training, a number of staff felt that the specific organizational context in which care planning occurs should have been factored into the course, especially around computer systems and assessment and care plan templates. This study adds to the existing literature on the constraining organizational factors upon care planning in which staff work (Bee *et al*. 2015a,b). Whilst previous research has shown that professionals recognize the importance of psychiatrists and senior management attending new training initiatives to drive the implementation of learning (Bee *et al*. 2015b), it was thus disappointing that no psychiatrists or senior management attended training.

### Strengths and limitations

The strengths of the study are that it has evaluated a new training programme on a key aspect of mental health clinical practice, based on a co‐production model. There was a high response rate to the TARS, with a large sample size, from participants from a wide variety of teams, professional roles and geographic areas. The integration of quantitative and qualitative findings allowed us to assess the overall acceptability ratings and valued aspects of the training as well as areas for improvement. This study adds to the literature on post‐registration, CPD training and the inclusion of service users and carers in training healthcare professionals.

The limitations of the study were the lack of demographic data for questionnaire participants, which made it difficult to assess response patterns across different demographic groups (e.g. different mental health professional roles). The acceptability and perceived impact ratings were assessed immediately after the two‐day training; thus, these data do not capture the final aspect of the training package, which consisted of six hours of clinical supervision per team. Therefore, we have limited data on the long‐term acceptability and impact, i.e. whether trainees seek to implement the training into practice. A further limitation is that the staff who attended the training may not be reflective of whole care teams, as is evidenced by the lack of psychiatrists and senior management in the training.

### Implications for practice

Mental health nurses using co‐production models of delivering training to other mental health professionals can be confident that such initiatives will be warmly welcomed, acceptable and impactful. On the basis of the results reported here, we encourage more use of this approach. Staff value the time and opportunity to reflect on their care planning practice in a training environment and to work collaboratively with colleagues. Further research is needed into the clinical effectiveness of this training in improving outcomes for service users and carers and this is being addressed in the EQUIP cluster randomized controlled trial.

## Relevance statement

This study has evaluated the acceptability of a new training programme on a key aspect of mental health clinical practice, based on a co‐production model. The study adds to the literature on CPD training and on co‐production models of training in particular and suggests that co‐facilitation models of service user involvement in training can be very impactful.
